# The Rate and Predictors of Allergic Fungal Rhinosinusitis Recurrence Post-sinus Surgery: A Retrospective Cohort Study

**DOI:** 10.7759/cureus.43398

**Published:** 2023-08-13

**Authors:** Naif Alfattani, Abdulmohsen S Alqurashi, Abdulrahman F Kabli, Aseel Haji, Bassam T Alharthi, Ammar K Mandili

**Affiliations:** 1 Otolaryngology-Head and Neck Surgery, Al-Noor Specialist Hospital, Makkah, SAU; 2 Medicine and Surgery, Umm Al-Qura University, Makkah, SAU; 3 Medicine, Umm Al-Qura University, Makkah, SAU; 4 Otolaryngology-Head and Neck Surgery, Aseer Central Hospital, Abha, SAU

**Keywords:** postoperative period, hypersensitivity, retrospective studies, chronic rhinosinusitis, allergic fungal rhinosinusitis

## Abstract

Objectives

Chronic rhinosinusitis (CRS) is the persistent inflammation of the mucosal lining of the paranasal sinuses (PNS). By definition, the inflammatory process persists beyond 12 weeks. One of its subtypes is allergic fungal rhinosinusitis (AFRS), which has a high risk of recurrence, leading to revision surgery. This study aimed to establish the predictive factors for the recurrence of AFRS in post-sinus surgery patients.

Methods

This single-center retrospective study was conducted in Al-Noor Specialist Hospital, Makkah, Saudi Arabia. The charts of patients with AFRS who underwent surgery in our rhinology clinic between 2000 and 2020 were reviewed.

Results

Among the 116 patients included in this study, approximately half (53%) were female, with a median age of 24.5 years. Thirty-nine (33.6%) patients had recurrence post-sinus surgery, with 33.3% occurring within six months of follow-up. The results showed that patients with coexisting bronchial asthma were three times more likely to experience recurrence (adjusted odds ratio {AOR}, 3.43; confidence interval {CI}, 1.35-8.71), patients with uncorrected deviated nasal septum (DNS) were three times more likely to experience symptoms again following surgery (AOR, 3.70; CI, 1.14-12.02), and patients who presented with concomitant sinus headaches are 66% less likely to experience recurrence (AOR, 0.34; CI, 0.13-0.86).

Conclusion

The results showed that 33.62% of patients experienced recurrence following surgery. Bronchial asthma and DNS were strongly associated with recurrence; however, their presence does not always imply the need for additional surgery.

## Introduction

Chronic rhinosinusitis (CRS) is the persistent inflammation of the mucosal lining of the paranasal sinuses (PNS) for at least 12 weeks with or without topical or systemic therapeutic measures [1]. It is classified into primary and secondary CRS. Primary CRS is furtherly categorized based on anatomical localization and the dominant endotype [2]. Allergic fungal rhinosinusitis (AFRS) is a type 2 dominant noninvasive disease, characterized by an intensified inflammatory process through immunoglobulin E (IgE)-mediated hypersensitivity against fungal components. The main pathologic feature of AFRS is the partial or complete obstruction of the ostia of the paranasal sinuses, which become packed with thick, heavy, and dense mucinous secretions accompanied by eosinophil degranulation and mast cell [3]. Five major criteria were proposed by Bent and Kuhn for the diagnosis of AFRS: (1) confirmed type 1 hypersensitivity by history, serology, or skin tests; (2) the presence of nasal polyps; (3) radiological findings on computed tomography (CT); (4) eosinophil-containing mucus; and (5) positive fungal stain [4].

The ideal management for AFRS is still under scrutiny. However, both surgical and medical interventions may be required [5]. The medical and surgical component thrust is to eradicate the allergic mucin and debris, establish aeration and permanent sinus drain, and control recurrence. In 2015, Philpott et al. [6] studied the incidence of revision surgery in the United Kingdom and found that patients with AFRS had high risks of both initial and revision surgeries. However, it must be noted that the overall prevalence of AFRS varies according to geographical location. A review of the literature revealed that large numbers of AFRS cases were reported from regions of elevated temperature and humidity [7]. An incidence of 6.95%-7.4% was reported in the Asian population based on a nationwide survey conducted in China and Korea [8]. Meanwhile, the rate was between 2.1% and 4.2% in Western countries [9].

Functional endoscopic sinus surgery (FESS) is commonly required for the management of AFRS. Approximately 54.5% of cases require revision surgery [10]. Surgeons may encounter further challenges while performing revision FESS due to the past removal of critical anatomical landmarks and the presence of adhesions. Lee et al. [11] compared the surgical outcomes of primary and revision endoscopic sinus surgeries and reported similar success rates. Although high recurrence rates have been reported, the risk factors and predictors of the disease have not yet been extensively investigated. The aim of this study is to establish predictive factors for recurrence in patients with AFRS post-sinus surgery.

## Materials and methods

Study design and selection of patients

This single-center retrospective cohort study was conducted in a tertiary hospital in Makkah, Saudi Arabia. The charts of patients who presented to our rhinology clinic with chronic sinusitis and underwent rhinology surgery from November 2000 to December 2020 were reviewed. Patients who met the following criteria for AFRS were included: the presence of eosinophilic mucin, nasal obstruction, unilateral or bilateral nasal polyp, and computed tomography (CT) results characteristic of AFRS. Patients who are immunocompromised, have uncontrolled diabetes, are histopathologically diagnosed with invasive fungal infection, underwent sinus surgery previously outside our hospital, underwent rhinology surgery other than functional endoscopic sinus surgery (FESS), or are followed up for less than nine months were excluded.

Data collection and assessment criteria

Medical records were screened, and data regarding sociodemographic information, clinical presentation, histopathological findings, past medical history, paranasal sinus (PNS) CT scan, pre- and postoperative medical management, intraoperative notes, and postoperative clinic visit notes were extracted.

Patients received nasal irrigation, antihistamine, intranasal steroid, and/or systematic steroid preoperatively. All the included patients underwent FESS, and turbinectomy or septoplasty was performed whenever indicated. Patients received saline nasal douching and were placed on intranasal steroids postoperatively. A total of 42 patients received systemic steroids with prednisone 0.2-0.5 mg/day for 5-7 days. Subsequently, they were followed up and reassessed at 1-2 weeks, three months, and 6-9 months postoperatively and as needed afterward. Patients were considered to have a recurrence if they developed at follow-up nasal obstruction, nasal discharge, postnasal drip, or loss of smell or if an endoscopic examination showed a nasal polyp or thick secretions. In addition, a PNS CT scan showed the opacification of the sinuses or double density.

Data analysis

The data were extracted, revised, cleaned, coded, and input into the statistical software Statistical Package for Social Sciences (SPSS) version 25 (IBM SPSS Statistics, Armonk, NY). All statistical analyses used two-tailed tests. Statistical significance was set at P < 0.05. Descriptive analysis based on frequency and percentage was performed for categorical variables. Since the numerical variables showed abnormal distribution, the median and interquartile ranges (IQR) were calculated. Comparisons between the groups were performed using chi-square test, Fisher’s exact test, and Mann-Whitney test whenever appropriate. Univariate regression analysis was performed to explore variables that predict recurrence post-surgery. Factors with P < 0.25 were included for the multivariate logistic regression analysis. In multivariate logistic regression, a backward stepwise approach was performed with a removal criterion of P ≥ 0.10. If the removed variables changed the model’s β coefficient by more than 20%, the variables were retained again in the model to adjust other variables in the model.

## Results

**Figure 1:**
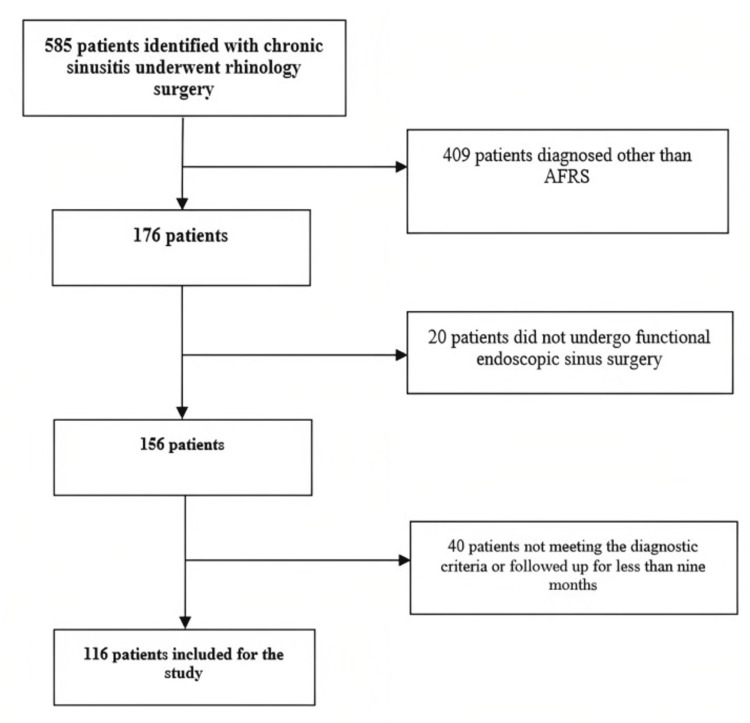
Flow chart of patient inclusion AFRS: allergic fungal rhinosinusitis

A total of 585 patients were diagnosed with chronic sinusitis and underwent surgery. Among them, 409 patients were excluded because they were diagnosed with other diseases other than AFRS. In addition, 20 patients were excluded because they did not undergo FESS. Moreover, 40 patients did not meet the diagnostic criteria or were followed up for less than nine months. Thus, 116 patients were finally included in our study as shown in Figure 1.

Regarding the characteristics of the patients with AFRS, approximately half of the patients were females, with ages ranging from 18.6 to 34 years and a median age of 24.5 years. The patients reported the following: nasal obstruction (88.8%), headache (70.7%), bilateral nasal polyp (52.6%), unilateral nasal polyp (47.4%), rhinorrhea (25%), and sneezing (19%). In addition, a significant number of patients were found to have a clinically significant deviated nasal septum (DNS) (12.9%). A total of 31 patients (26.7%) had a history of bronchial asthma, while the remaining patients had eczema (12.1%), diabetes mellitus (6.9%), and hypothyroidism (0.9%). FESS was performed on all 116 patients; in addition, 16.4% had turbinectomy, and 13.8% had septoplasty (Tables 1, 2).

**Table 1:** Baseline characteristics for the cohort of patients with allergic fungal rhinosinusitis †Positive fungi in gram stain and/or culture PND, postnasal discharge; DNS, deviated nasal septum; COM, chronic otitis media; IQR, interquartile range

Parameter	Overall (n = 116)	Non-recurrence (n = 77)	Recurrence (n = 39)	P-value
Female gender, N (%)	62 (53.4)	43 (55.8)	19 (48.7)	0.56
Age at diagnosis, median (IQR)	24.5 (18.6-34)	24 (19-34)	26 (16-34)	0.55
Clinical presentation and findings				
Nasal obstruction, N (%)	103 (88.8)	68 (88.3)	35 (89.7)	1.0
Headache, N (%)	82 (70.7)	58 (75.3)	24 (61.5)	0.14
Bilateral nasal polyp, N (%)	61 (52.6)	42 (54.5)	19 (48.7)	0.56
Unilateral nasal polyp, N (%)	55 (47.4)	35 (45.5)	20 (51.3)	0.56
Rhinorrhea, N (%)	29 (25.0)	19 (24.7)	10 (25.6)	1.0
Sneezing, N (%)	22 (19.0)	12 (15.6)	10 (25.6)	0.22
PND, N (%)	15 (12.9)	11 (14.3)	4 (10.3)	0.58
DNS, N (%)	15 (12.9)	6 (7.8)	9 (23.1)	0.037
Nasal turbinate hypertrophy, N (%)	14 (12.1)	7 (50)	7 (50)	0.23
Proptosis of the eyes, N (%)	11 (9.5)	8 (10.4)	3 (7.7)	0.75
Positive fungal†, N (%)	16 (13.8)	10 (13.0)	6 (15.4)	0.78
Diplopia, N (%)	3 (2.6)	1 (1.3)	2 (5.1)	0.26
COM, N (%)	1 (0.9)	1 (1.3)	0 (0)	1
Total Lund-Mackay score, median (IQR)	16 (12-22)	16 (12-22)	18 (12-24)	0.18
Past medical history				
Bronchial asthma, N (%)	31 (26.7)	16 (20.8)	15 (38.5)	0.049
Eczema, N (%)	14 (12.1)	8 (10.4)	6 (15.4)	0.55
Diabetes mellitus, N (%)	8 (6.9)	7 (9.1)	1 (2.6)	0.26
Hypothyroidism, N (%)	1 (0.9)	0 (0)	1 (100)	0.34

**Table 2:** Baseline characteristics for the cohort of patients with allergic fungal rhinosinusitis in operative management FESS: functional endoscopic sinus surgery

Parameter	Overall (n = 116)	Non-recurrence (n = 77)	Recurrence (n = 39)	P-value
FESS, N (%)	116 (100)	77 (100)	39 (100)	-
Septoplasty, N (%)	16 (13.8)	10 (13)	6 (15.4)	0.78
Turbinectomy, N (%)	19 (16.4)	13 (16.9)	6 (15.4)	1

Out of 116 patients, 39 had a recurrence. The presence of DNS (P = 0.037) and a history of bronchial asthma (P = 0.049) were significantly associated with the recurrence of symptoms after surgery. A significant association between patients with other clinical findings and recurrence was not observed as shown in Table 1.

A total of 13 patients (33.3%) experienced recurrence within six months postoperatively; 10 patients (25.6%) experienced recurrence between seven months and one year postoperatively; 10 patients (25.6%) experienced recurrence beyond two years postoperatively. Among these, 17 (43.6%) underwent revision surgery, while 22 (56.4%) did not. Five patients (29.4%) had revision surgery within six months; four patients (23.5%) had revision surgery between seven and 12 months; three patients (17.6%) had revision surgery between 13 and 18 months; one patient (5.9%) had revision surgery between 19 and 24 months; four patients (23.5%) had revision surgery after more than two years (Table 3).

**Table 3:** Univariate description of the recurrence of otolaryngological symptoms post-surgical management

Parameter	Value
Recurrent of otolaryngological symptoms post-surgery, n (%)	
No	77 (66.4)
Yes	39 (33.6)
Duration from post-surgery to recurrence (N = 39), n (%)	
0-6 months	13 (33.3)
7-12 months	10 (25.6)
13-18 months	3 (7.7)
19-24 months	3 (7.7)
>24 months	10 (25.6)
Underwent revision surgery (N = 39), n (%)	
No	22 (56.4)
Yes	17 (43.6)
Duration from surgery to revision surgery (N = 17), n (%)	
0-6 months	5 (29.4)
7-12 months	4 (23.5)
13-18 months	3 (17.6)
19-24 months	1 (5.9)
>24 months	4 (23.5)

Regarding the results of the univariate of patient characteristics in predicting post-surgery recurrence, female gender was not a significant predictor of recurrence (odds ratio {OR}, 0.75; confidence interval {CI}, 0.35-1.50), as well as age (OR, 0.99; CI, 0.95-1.02). Patients with DNS were three times more likely to experience a recurrence than patients without DNS (OR, 3.55; CI, 1.16-10.86). In addition, patients with nasal obstruction had a marginally increased likelihood of experiencing a recurrence (OR, 1.16; CI, 0.33-4.03). The presence of bilateral nasal polyp (OR, 0.79; CI, 0.37-1.71), unilateral nasal polyp (OR, 1.26; CI, 0.58-2.73), headache (OR, 0.52; CI, 0.23-1.20), fungus (OR, 1.22; CI, 0.41-3.64), and rhinorrhea (OR, 1.05; CI, 0.43-2.55) was not significant predictors of recurrence. Patients with a history of bronchial asthma were twice as likely to develop symptoms again (OR, 2.38; CI, 1.02-5.56). Patients with diabetes mellitus (OR, 0.26; CI, 0.03-2.22) and eczema (OR, 1.57; CI, 0.50-4.89) and who underwent septoplasty (OR, 1.22; CI, 0.41-3.64) or turbinectomy (OR, 0.90; CI, 0.31-2.57) were not significantly associated with recurrence (Tables 4, 5).

**Table 4:** Univariate and multivariate analysis of patient characteristics in predicting post-surgery recurrence OR, odds ratio; AOR, adjusted odds ratio; CI, confidence interval; PND, postnasal discharge; DNS, deviated nasal septum; IQR, interquartile range

Parameter	Univariate		Multivariate	
	OR	95% CI	AOR	95% CI
Female sex	0.75	0.35-1.63	-	-
Age at diagnosis (years)	0.99	0.95-1.02	-	-
Clinical presentation and findings				
Nasal obstruction	1.16	0.33-4.03	-	-
Headache	0.52	0.23-1.20	0.34	0.13-0.86
Bilateral nasal polyp	0.79	0.37-1.71	-	-
Unilateral nasal polyp	1.26	0.58-2.73	-	-
Headache	0.52	0.23-1.20	0.34	0.13-0.86
Rhinorrhea	1.05	0.43-2.55	-	-
Sneezing	1.87	0.73-4.81	-	-
PND	0.69	0.20-2.31	-	-
DNS	3.55	1.16-10.86	3.70	1.14-12.02
Nasal turbinate hypertrophy	2.19	0.71-6.76	3.32	0.92-11.99
Proptosis of the eyes	0.72	0.18-2.88	-	-
Presence of fungus	1.22	0.41-3.64	-	-
Diplopia	4.11	0.36-46.78	-	-
Total Lund-Mackay score, median (IQR)	1.04	0.97-1.11	-	-
Past medical history				
Bronchial asthma	2.38	1.02-5.56	3.43	1.35-8.71
Eczema	1.57	0.50-4.89	-	-
Diabetes mellitus	0.26	0.03-2.22	-	-

**Table 5:** Univariate and multivariate analysis of operative management in predicting post-surgery recurrence OR, odds ratio; CI, confidence interval

Parameter	Univariate		Multivariate	
	OR	95% CI	OR	95% CI
Septoplasty	1.22	0.41-3.64	-	-
Turbinectomy	0.90	0.31-2.57	-	-

Multivariate analysis was performed to include variables that showed significance at the univariate analysis. The final model included headache, DNS, nasal turbinate hypertrophy, and bronchial asthma. As shown in Table 4, patients with bronchial asthma were three times more likely to experience recurrence (adjusted odds ratio {AOR}, 3.43; CI, 1.35-8.71), as well as patients with DNS (AOR, 3.70; CI, 1.14-12.02). Patients who presented with headaches were 66% less likely to experience recurrence (AOR, 0.34; CI, 0.13-0.86). Meanwhile, patients with nasal turbinate hypertrophy insignificantly had a threefold risk of recurrence (AOR, 3.32; CI, 0.92-11.99).

## Discussion

The clinical features of AFRS and other forms of chronic sinusitis highly overlap [12]. The symptoms are initially similar to CRS; however, as AFRS progresses, a unique set of radiological and pathophysiological findings can be observed. The specific pathogenesis of AFRS has not yet been fully understood. Nevertheless, it is unilateral in 19% of cases; however, there is no clear explanation for why AFRS tends to be unilateral [13].

The main symptoms of patients with AFRS in our study were nasal obstruction and headache. This finding is similar to another study that was conducted from January 2011 to December 2015 at the Liaquat National Hospital, which reported that the common presenting symptoms of AFRS included nasal obstruction, headache, nasal discharge, and proptosis [14].

After nasal obstruction and headache, rhinorrhea was the most common complaint of patients. This result was also supported by a study conducted by the honorary and corresponding members (otorhinolaryngologists) of the Italian Society of Rhinology [15].

Although the recurrence rate of AFRS varies from one study to another, it was reportedly mostly high (e.g., in our study, 33% of patients had a recurrence). These results are almost consistent with the study of Alghonaim et al. [16], which reported that 29% of patients with AFRS had recurrence post-sinus surgery. Moreover, a study by Makary et al. [13] reported a recurrence rate of up to 50% in patients with AFRS. In addition, a previous study published in 2017 by Marglani et al. [17] included 52 patients diagnosed with AFRS. Hence, only 16 patients reported to have unilateral disease involvement had a recurrence rate of 19%, and those with bilateral AFRS involvement had a 61% recurrence. These findings should be taken into consideration in managing these patients.

In the current study, asthma and AFRS were strongly associated. This finding was also supported by a previous study in Karachi, Pakistan, from December 2016 to November 2018, which reported that patients with asthma are more likely to have this disease [18]. In addition, patients with concha bullosa usually develop AFRS, which means that they may be potentially associated [13].

In summary, our study determined the recurrence rate and potential factors that may lead to revision surgery in patients with AFRS. Nevertheless, our study has several limitations. The most significant limitations were the retrospective nature of the study and the inclusion of only a small number of patients. Moreover, the possibility of our patients reporting facial pain and heaviness as headache was also high. Another weak point was the lack of the standardization of the operative technique and postoperative care. In addition, the follow-up period was limited to only nine months. Finally, due to the varying culture protocols of different laboratories, reporting on fungal cultures was challenging.

## Conclusions

A total of 116 patients were diagnosed with AFRS, with 39 experiencing a recurrence but only 17 undergoing revision surgery. Bronchial asthma and DNS were strongly associated with recurrence; however, their presence does not always imply the need for additional surgery, as this decision must always depend on the treating surgeon and postoperative care. Nevertheless, further understanding of the establishment of AFRS in poorly aerated nasal passages and the associated poorly aerated sinuses is a factor that must be borne in mind.
